# Vaspin Mediates the Intraorgan Crosstalk Between Heart and Adipose Tissue in Lipoatrophic Mice

**DOI:** 10.3389/fcell.2021.647131

**Published:** 2021-09-24

**Authors:** Donghui Zhang, Hong Zhu, Enbo Zhan, Fan Wang, Yue Liu, Wei Xu, Xian Liu, Jingjin Liu, Shufeng Li, Yong Pan, Yongshun Wang, Wei Cao

**Affiliations:** ^1^Department of Cardiology, The Second Affiliated Hospital of Harbin Medical University, Harbin, China; ^2^The Key Laboratory of Myocardial Ischemia, Chinese Ministry of Education, Harbin, China; ^3^Department of Geriatrics, Xuanwu Hospital, Capital Medical University, Beijing, China; ^4^Department of Cardiology, The First Affiliated Hospital of Harbin Medical University, Harbin, China; ^5^Department of Cardiology, The Fourth Affiliated Hospital of Harbin Medical University, Harbin, China; ^6^Department of Cardiology, Shenzhen People’s Hospital (The Second Clinical Medical College, Jinan University, The First Affiliated Hospital, Southern University of Science and Technology), Shenzhen, China; ^7^Department of Pathophysiology, Shenzhen University, Shenzhen, China; ^8^Guangdong Provincial Key Laboratory of Genome Stability and Disease Prevention, Shenzhen, Guang Dong, China

**Keywords:** lipoatrophy, adipose tissue, vaspin, cardiomyopathy, mitochondria, AKT

## Abstract

Lipoatrophy is characterized as selective loss of adipose tissues, leading to the severity of cardiovascular disorders. Therefore, there was close intraorgan crosstalk between adipose tissue and cardiovascular in lipoatrophy. A-ZIP/F-1 mouse, a well-established lipoatrophic model, and primary cardiomyocytes were used for investigating the pathophysiological changes and molecular mechanisms. A-ZIP/F-1 mice had severe fat loss and impaired ventricular function during growth, but closely associated with the reduction of circulating vaspin levels. Administration of recombinant vaspin protein improved cardiac structural disorders, left ventricular dysfunction, and inflammatory response in lipoatrophic mice. In detail, vaspin decreased cardiac lipid deposits, but enhanced mitochondrial biogenesis and activities. Interestingly, A-ZIP/F-1 mice transplanted with normal visceral adipose tissues exhibited improvement in cardiac structural remodeling and mitochondrial function. Mechanistically, vaspin increased cardiac AKT activity, which guaranteed the mitochondrial benefits of vaspin in lipoatrophic mice and primary mouse cardiomyocytes. The present study suggested that vaspin possessed biological benefits in attenuating lipoatrophy-induced cardiomyopathy onset, and targeting vaspin/AKT signaling was a potential strategy to maintain heart metabolism.

## Introduction

Lipoatrophy, characterized by fat loss, is accompanied with severe hyperlipidemia, insulin resistance, and peripheral tissue damages. One of key phenotypic features that frequently occurred is cardiomyopathy, which exhibits dyslipidemia and then cardiac pathological remodeling (Ishii et al., 1989). Higher levels of lipid deposit contributes to cardiac lipotoxicity, accompanied with excess inflammatory response (Sekhar et al., 2004). Therefore, it is necessary to explore an effective approach to combat lipoatrophy-induced cardiac injuries.

Adipose tissue, as a major storage site of neutral lipids, is integrally involved in a variety of metabolic homeostasis including serum lipid profiles, insulin sensitivity, and glucose disposal activity (Janssen et al., 2002). The elevated fat mass is an independent risk factor for multiple metabolic complications (Kissebah et al., 1982). However, lipoatrophy, with selective fat loss, also leads to severe metabolic syndrome in both humans and rodents (Leow et al., 2003). Emerging studies have supported adipocytes, which can be identified as critical endocrine cells, which secrete amounts of factors named adipokines. These adipokines participate in complex networks of metabolic cardiovascular disease. For example, adiponectin improved dietary- or ischemia/perfusion-induced cardiac injuries (Shibata et al., 2004, 2005). In lipodystrophic patients, the serum levels of adiponectin were extremely lower (Haque et al., 2002). In human immunodeficiency virus (HIV)-induced lipoatrophy patients, administration of HIV protease inhibitors could increase circulating levels of adiponectin (Lee et al., 2004). In contrast, another toxic adipokine, adipocyte fatty acid-binding protein (A-FABP), suppressed cardiomyocyte contraction and cardiac function (Lamounier-Zepter et al., 2009; Zhou et al., 2015). These previous studies implied that adipokines may bridge adipose tissues and nearby tissues, and targeting adipokines was a potential therapeutic strategy for alleviating lipoatrophy-induced cardiovascular disorders.

Vaspin, named visceral adipose tissue-derived serine protease inhibitor, was identified as an insulin-sensitizing adipokine in obesity (Hida et al., 2005). Recently, emerging evidence had implied that vaspin is involved in the process of multiple cardiovascular diseases. In patient with coronary artery disease, circulating levels of vaspin were significantly decreased and negatively related to disease severity (Kadoglou et al., 2011). Independent of insulin sensitivity, circulating level of vaspin was also a potential risk factor of carotid atherosclerosis (Esaki et al., 2014). Zhang et al. (2016) also showed that patients with acute myocardial infarction had lower levels of serum vaspin. Furthermore, administration of vaspin protected against multiple mouse cardiac injuries, including cardiomyocyte apoptosis, myocardial ischemia/reperfusion injury, and diabetic cardiomyopathy (Ke et al., 2018; Yuan et al., 2018; Li et al., 2019). Although it was discovered that vaspin possessed cardioprotective benefits, its role in lipoatrophy-induced cardiovascular disorders was unknown.

The present study identified whether adipose tissue-derived vaspin had therapeutic benefits in protecting against lipoatrophy-induced cardiomyopathy and investigated the possible molecular mechanisms. Through utilizing the A-ZIP/F1 genetic lipoatrophic mouse model, the present study determined that vaspin exhibited cardioprotective benefits in lipoatrophy, which facilitated the further clinical application of vaspin in combating lipoatrophy-related cardiomyopathy.

## Materials and Methods

### Animal Study

All mouse experimental protocols were approved by the Institutional Animal Care and Use Committee guidelines of Harbin Medical University. Male A-ZIP/F-1 mice [FVB-Tg(AZIP/F)1Vsn/J] was obtained from the Jackson Laboratory and then backcrossed with C57BL/6J mice four times to generate A-ZIP/F-1 mice with C57BL/6 background. After weaning, male A-ZIP/F-1 mice on the FVBxC57BL/6J background, aged 4 weeks, were fed with standard chow. The cardiac functional parameters were measured by echocardiography (Vevo 2100) after mice were fed at 4, 12, and 24 weeks. The mouse fat mass was measured by nuclear magnetic resonance (Bruker). Aged 16–24 weeks C57BL/6J mice were used as controls.

For vaspin treatment, aged 16-week male A-ZIP/F-1 mice were administrated with 100 μg/mouse recombinant vaspin protein (Z03199, GenScript) or PBS by osmatic pumps (1002, ALZET) as reported in a previous study (Sato et al., 2018). Once every 4 weeks, the mini-pumps were implanted subcutaneously. After treatment for 8 weeks, the mice were sacrificed, and the tissues were collected for further analysis.

For fat implantation, 16-week A-ZIP/F-1 mice were subcutaneously transplanted with 0.9 g epididymal fat from C57BL/6J mice for 8 weeks as reported in a previous study (Gavrilova et al., 2000). For administration with vaspin inhibitor, mice were locally injected with 1 × 10^9^ lentivirus-encoding vaspin siRNA (sc-76890, Santa Cruz) or control siRNA for 8 weeks. The vaspin siRNA or control were constructed into pLKO.1 puro vector with adipose tissue-specific *Fabp4*-promoter and mixed with lentivirus-packaging plasmids, including VSV, REV, and GAG for lentivirus generation. After 8 weeks of intervention, the mice were sacrificed, and the hearts were collected for further analysis.

### Echocardiographic Measurement

An echocardiography system (vevo 2100) was used to measure mouse cardiac function. Mice were anesthetized with 1–1.5% isoflurane. Left ventricular dimensions, including left ventricular LVAW, LVID, or LVPW thickness, were measured by M-mode echocardiography, and cardiac functional parameters, including EF, FS, and LV mass, were calculated. All calculated results were the mean of five consecutive cardiac cycles.

### Histological Staining of Cardiac Section

Left ventricular tissue was fixed in 4% paraformalin and embedded in paraffin. Five-micrometer paraffin sections were stained with hematoxylin and eosin (H&E) solution (ab245880, Abcam) or Sirius red staining reagents (ab150681, Abcam). Image analysis software (ImageJ) was used to measure average cardiomyocyte size and cardiac collagen contents. To examine mitochondrial morphology, stained heart sections were analyzed by an electronic microscope. The lipid droplets per field were represented by at least five fields.

### Lipid Analysis

The cardiac lipids were extracted with methanol/chloroform (1:2), dried in an evaporating centrifuge, and resuspended in 5% fat-free bovine serum albumin (BSA, 05470, Sigma) was extracted as reported in a previous study (Pan et al., 2014). The levels of triglyceride (T2449, Sigma) and free fatty acid (MAK044, Sigma) in mouse serum and hearts were measured by biochemical kits (Sigma Diagnostics).

### Measurement of Cardiac Mitochondrial Activity

Mouse cardiac or primary mouse cardiomyocyte mitochondrial parameters, including ATP, endogenous basal oxygen consumption, citrate synthase activity, and complex I activity, were measured as previous reports (Nie et al., 2018a). In brief, ATP levels were measured using an ATP measurement kit (Molecular Probes, Carlsbad, CA, United States), while endogenous basal oxygen consumption was measured with a Clark electrode in a water-jacketed chamber connected to a circulating water bath (Hansatech, Norfolk, United Kingdom). The citrate synthase activity was analyzed using a commercial measurement kit (Abcam). The activity of the electron transfer chain (ETC) complex I (NADH:ubiquinone reductase) was measured and expressed as a ratio to citrate synthase activity to account for mitochondrial enrichment.

### Enzyme-Linked Immunosorbent Assay Analysis

The TNF-a and IL-6 levels in the serum and homogenized cardiac tissues were determined with an enzyme-linked immunosorbent assay (ELISA) kit (88-7324-22 or 88-7064-22, Invitrogen, CA, United States) (Fang et al., 2015). Vaspin level was measured by using a kit from RayBiotech (EIAM-VAP-1, RayBio, GA, United States) according to the instructions of the manufacturer. The cardiac concentration of analyzed protein was normalized to the protein concentration of lysates.

### *In vitro* Experiments

Primary neonatal mouse cardiomyocytes were isolated by using a method published previously (Pan et al., 2014). Cells were cultured in RPMI 1640 medium. The siRNA encoding Akt was purchased from Cell Signaling Technology, and constructed into a pLKO.1 puro vector. For transfection with lentivirus-encoding si*Akt*, 1 10^6^ primary cardiomyocytes were incubated with 1 10^5^ viral particles for 48 h, then treated with recombinant vaspin protein (2.5 μg/ml) for 24 h.

### Immunoblot Analysis

Fifty micrograms of protein from hearts or cardiomyocytes was ran and separated by 10% SDS gel and electrotransferred to PVDF membranes. Each membrane was preblocked in 10% non-fat milk dissolved in solution (Tris-buffered saline, pH 7.6, containing 0.05% tween 20) and incubated with specific primary antibodies, including ANP (PA5-29559, thermo), BNP (PA5-29559, thermo), TGF-β1 (ab215715, Abcam), SMAD3 (ab40854, Abcam), α-porin (ab14734, Abcam), PGC-1α (ab176328, Abcam), p-AKT (4060, Cell Signaling Tech), AKT (4691, Cell Signaling Tech), p-GSK3β (9323, Cell Signaling Tech), GSK3β (9315, Cell Signaling Tech), and Tubulin (2128, Cell Signaling Tech). Immunoreactive bands were then detected by incubating the membrane with secondary antibodies conjugated to horseradish peroxidase (Cell Signaling Technology) and visualized using enhanced chemiluminescence reagents (Bio-Rad, Hercules, CA, United States). The relative protein expression was measured by using the ImageJ software (Image 1.38e) and normalized with the expression of the respective tubulin.

### Real-Time PCR Analysis

Cardiac tissues or cardiomyocytes were homogenized in TRIZOL (Invitrogen, Carlsbad, CA, United States) for extraction of RNA according to the protocol of the manufacturer. Five hundred nanograms of RNA was processed in reverse transcription and quantitative PCR by using Promega kits (Promega, Madison, WI, United States). The ABI Prism 7900 Sequence Detection System (Applied Biosystems, Alameda, CA, United States) was used for real-time qPCR analysis. The detected primers were synthesized from Invitrogen (Invitrogen, Shanghai, China), and the primer sequences were listed as follows: *pgc-1*α: F-5′-ACATCGCAATTCTCCCTT-3′; R-5′-CTC TTGAGCCTTTCGTGCTC-3′, *Nrf1*: F-5′-TTGGAACAGCAGT GGCAAGA-3′; R-5′-CTCACTTGCTGATGTATTTACTTCCAT-3′, *Nrf2*: F-5′-GCTTTTGGCAGAGACATTCC-3′; R-5′-ATCAG CCAGCTGCTTGTTTT-3′, *Tfam*: F-5′-AAGGGAATGGGAAA GGTAGA-3′; R-5′-AACAGGACATGGAAAGCAGAT-3′, *GAP DH*: F-5′-AGGAGCGAGACCCCACTAAC-3′; R-5′-GATGACC CTTTTGGCTCCAC-3′. The relative expression of each gene was normalized to the amount of *GAPDH*.

### Akt Kinase Activity Assay

Hearts from different groups were weighed, homogenated, and centrifuged. Supernatants were collected for further analysis. Phosphorylated-Akt activity in cardiac homogenates were determined by using a solid-phase sandwich ELISA according to the instructions of the manufacturer (PEL-GSK3b-S9-T-1, Raybiotech).

### Statistics Analysis

Data were represented as mean ± SEM. The Students’ *t*-test was used for comparing two groups, and one-way ANOVA was used for comparing multiple groups. The posttests were performed after ANOVA to correct for multiple testing. The statistical significance was calculated by using GraphPad Prism 8 (GraphPad, San Diego, CA, United States). A value of *p* < 0.05 was considered as a significant difference.

## Results

### The Lipoatrophy-Induced Cardiac Dysfunction Is Closely Associated With Plasma Level of Vaspin in A-ZIP/F-1 Mice

**FIGURE 1:**
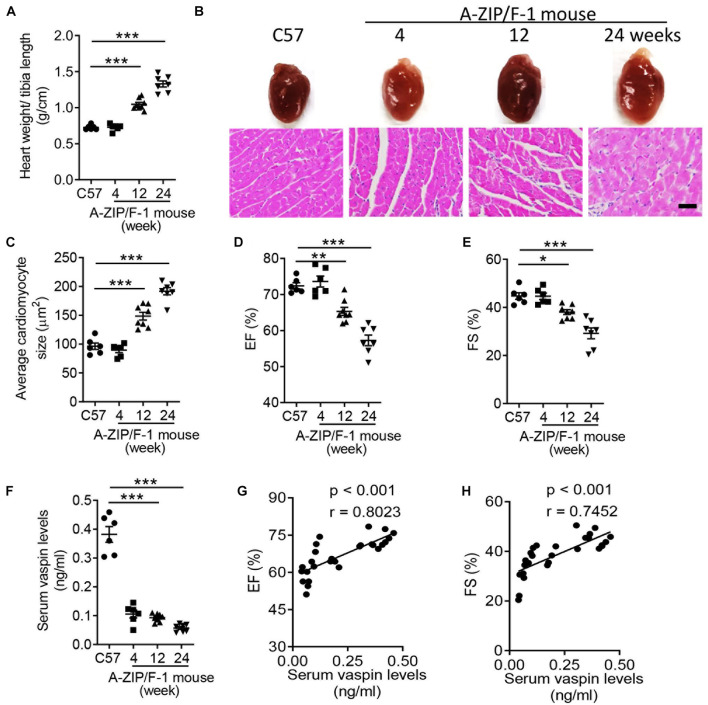
Cardiac dysfunction is negatively correlated with serum level of vaspin in A-ZIP/F-1 lipoatrophic mice. **(A)** The ratio of heart weight/tibia length of 4-, 12-, and 24-week-old A-ZIP/F-1 mice. The C57BL/6J (C57) mice were the control group. **(B,C)** Representative images of whole hearts and cardiac slides were stained with hematoxylin and eosin solution **(B)**, and quantitative analysis of average cardiomyocyte size **(C)**. Scale bar = 20 μm. **(D,E)** Echocardiographic analysis of mouse cardiac ejection fraction (EF) **(D)** and fractional shortening (FS) **(E)**. **(F)** Enzyme-linked immunosorbent assay (ELISA) analysis of mouse serum levels of vaspin. **(G,H)** The correlation between serum levels of vaspin and EF and FS in A-ZIP/F-1 mice. Results are shown as mean ± SEM, and *n* = 6–8 mice/group. *p < 0.05, **p < 0.01, ***p < 0.001.

Lipoatrophy, characterized by loss of adipose tissues, contributed to severe metabolic disorders (Reitman et al., 2000). One of the metabolic complications was cardiomyopathy, which was described in multiple clinical trials (Bhayana et al., 2002; Van Maldergem et al., 2002; Caux et al., 2003). In patients with generalized congenital lipoatrophy, the incidence of left ventricular (LV) hypertrophy was higher (Bhayana et al., 2002; Van Maldergem et al., 2002). A-ZIP/F-1 mouse was a well-established experimental model for exploring the consequences of lipoatrophy (Moitra et al., 1998). However, it was unclear how lipoatrophy modulated cardiac remodeling in A-ZIP/F-1 mice. Then, we fed the A-ZIP/F-1 mice with chow diet, and C57BL/6J (C57) mice were used as control. As showed in Figure 1A, the ratio of heart weight and tibia length was time-dependently increased, whereas fat mass percentage was significantly decreased in A-ZIP/F-1 mice (Supplementary Figure 1A). There was enlarged cardiac morphology during the 24-week growth, and the cardiac histological staining also found that the cardiomyocyte size was also enlarged in old mice (Figures 1B,C). Furthermore, echocardiographic analysis showed that cardiac functional parameters, including ejection fraction (EF) and fractional shortening (FS), were significantly decreased in old A-ZIP/F-1 mice (Figures 1D,E). Meanwhile, both triglycerides (Supplementary Figure 1B) and free fatty acid (Supplementary Figure 1C) were remarkably increased in A-ZIP/F-1 mouse hearts, compared with wild-type C57BL/6J mice. All these results supported that A-ZIP/F-1 mice exhibited severe cardiac hypertrophy and dysfunction.

Vaspin had protective benefits in the process of several cardiac injuries (Yuan et al., 2018; Li et al., 2019), but it was unknown whether vaspin participated in the lipoatrophy-induced cardiomyopathy. To this end, we measured the serum vaspin levels in A-ZIP/F-1 and compared C57 mice. The circulating levels of vaspin were obviously decreased in lipodystrophic mice, compared with C57 mice (Figure 1F, *p* < 0.001). More importantly, serum level of vaspin was positively correlated with cardiac EF (Figure 1G, *r* = 0.8023, *p* < 0.001) and FS (Figure 1H, *r* = 0.7452, *p* < 0.001). These findings indicated that vaspin might have cardiac protective effects in lipodystrophic A-ZIP/F-1 mice.

### Administration of Vaspin Recombinant Protein Improves Cardiac Structural Disorders, Inflammatory Response, and Mitochondrial Dysfunction in Lipoatrophic A-ZIP/F-1 Mice

**FIGURE 2:**
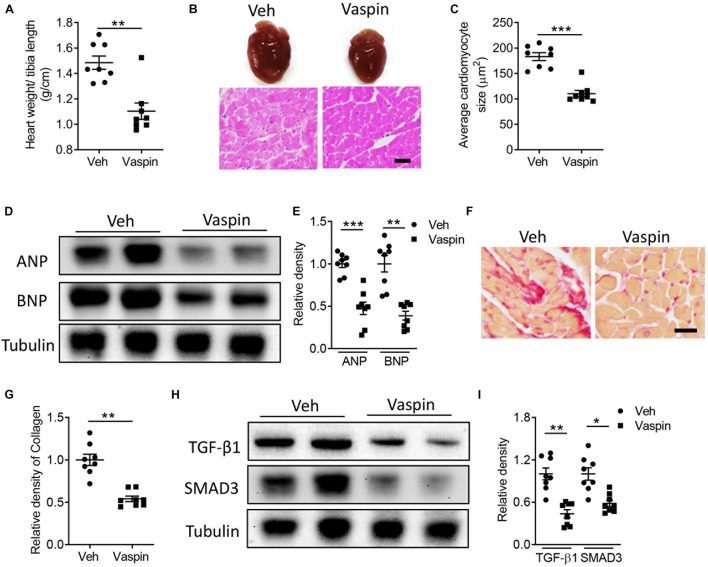
Administration of recombinant vaspin protein attenuates cardiac hypertrophy and fibrosis in A-ZIP/F-1 lipoatrophic mice. Sixteen-week-old A-ZIP/F-1 mice were treated with recombinant vaspin protein (100 μg/mouse) by an osmatic pump for 8 weeks. **(A)** The ratio of heart weight/tibia length. **(B,C)** Representative images of whole hearts, and cardiac slides were stained with hematoxylin and eosin solution **(B)**, and quantitative analysis of average cardiomyocyte size **(C)**. Scale bar = 20 μm. **(D,E)** Western blot analysis of cardiac atrial natriuretic peptide (ANP) and B-type natriuretic peptide (BNP) **(D)**, and quantitative analysis of relative density of ANP/tubulin and BNP/tubulin **(E)**. **(F,G)**. Cardiac slides were stained with Sirius red solution **(F)** and quantitative analysis of relative density of collagen **(G)**. Scale bar = 40 μm. **(H,I)** Western blot analysis of cardiac TGF-β1 and SMAD3 **(H)** and quantitative analysis of relative density of TGF-β1/tubulin and SMAD3/tubulin **(I)**. Results are shown as mean ± SEM, and *n* = 8 mice/group. *p < 0.05, **p < 0.01, ***p < 0.001.

To delineate the potential effect of vaspin in lipoatrophy-induced cardiac injuries, we next administrated the A-ZIP/F-1 mice with vaspin recombinant protein by using an osmatic pump. Aged 16-week A-ZIP/F-1 mice were treated with vaspin recombinant protein for another 8 weeks. As shown in Supplementary Figure 2A, mouse serum vaspin concentration was quickly increased after implantation of an osmatic pump and kept at more than fourfold to normal level (*p* < 0.001). Meanwhile, cardiac vaspin level was also significantly increased in vaspin recombinant protein-treated mice (Supplementary Figure 2B). Administration of vaspin improved cardiac hypertrophic parameters, including reduction of the ratio of heart weight/tibia length (Figure 2A), and cardiac and cardiomyocyte size (Figures 2B,C, *p* < 0.001). Atrial natriuretic peptide (ANP) and B-type natriuretic peptide (BNP) were major markers of cardiac hypertrophy (Nishikimi et al., 2006). Immunoblot analysis found that vaspin could significantly decrease the protein expression of ANP and BNP, compared with vehicle (Veh)-treated A-ZIP/F-1 mice (Figures 2D,E). Furthermore, as Table 1 showed, administration of vaspin increased the values of cardiac EF (*p* < 0.01) and FS (*p* < 0.01) in lipodystrophic mice. Besides, vaspin also suppressed the abnormal enlargement of left ventricle, including reduction of LVAW, LVID, or LVPW thickness (*p* < 0.01). The calculated vaspin-treated mouse left ventricle mass was also decreased to 75% of Veh-treated A-ZIP/F-1 mice (*p* < 0.001). Abnormal fibrotic formation was another key character in cardiac remodeling (Travers et al., 2016). The Sirius Red staining showed that vaspin suppressed the collagen deposits in hearts (Figures 2F,G). Mechanistically, vaspin also inhibited the fibrotic signaling, including downregulation of cardiac TGF-β1 and SMAD3 levels (Figures 2H,I).

**TABLE 1:** Echocardiography analysis of mice treated with vaspin or control vehicle.

Variables	Veh	Vaspin	*p-*value
Heart rate (beats/min)	475 ± 23	446 ± 18	ns
EF%	63.12 ± 11.32	75.32 ± 7.23	<0.01
FS%	37.32 ± 5.12	49.32 ± 6.12	<0.01
LVAW;d (mm)	1.29 ± 0.32	0.91 ± 0.22	<0.01
LVID;d (mm)	4.31 ± 0.67	3.59 ± 0.32	<0.01
LVPW;d (mm)	1.15 ± 0.11	0.84 ± 0.14	<0.001
LVAW;s (mm)	1.67 ± 0.34	1.27 ± 0.23	<0.01
LVID;s (mm)	2.67 ± 0.53	1.89 ± 0.21	<0.001
LVPW;s (mm)	1.57 ± 0.36	1.11 ± 0.31	<0.01
LV mass (mg)	149.54 ± 15.43	110.34 ± 14.32	<0.001

*Data are means ± SEM. EF, ejection fraction; FS, fractional shortening; d, diastolic; s, systolic; LVAW, left ventricular end anterior wall thickness; LVID, left ventricular internal dimension; LVPW, left ventricle posterior wall thickness; LV mass, left ventricle mass.*

Excessive cardiac inflammation exaggerated the cardiac structural and functional disorders (Pan et al., 2013, 2014). Then, we measured the levels of inflammatory cytokines in hearts. As shown in Supplementary Figures 2C,E, administration of vaspin effectively decreased the protein levels of cardiac TNF-α (Supplementary Figure 2C, *p* < 0.001), IL-1β (Supplementary Figure 2D, *p* < 0.01), and nitric oxide (Supplementary Figure 2E, *p* < 0.01). These results indicated that vaspin had the anti-inflammatory effects in lipoatrophy-induced cardiac injuries.

**FIGURE 3:**
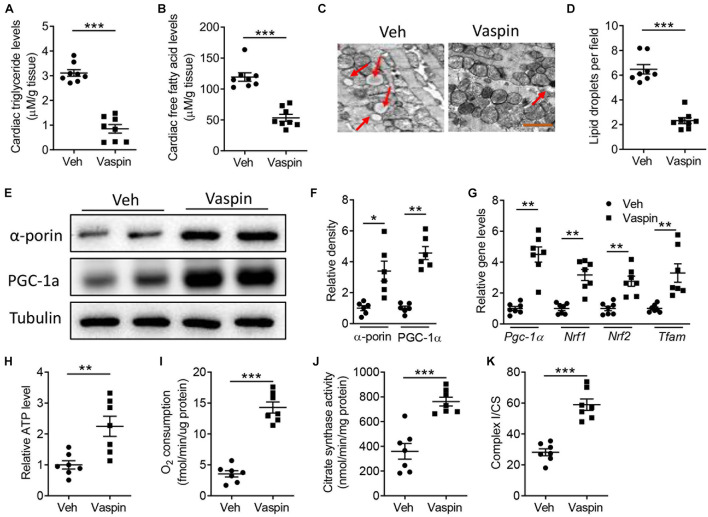
Vaspin increases cardiac mitochondrial biogenesis and activities in A-ZIP/F-1 lipoatrophic mice. Sixteen-week-old A-ZIP/F-1 mice were treated with recombinant vaspin protein (100 μg/mouse) by an osmatic pump for 8 weeks. **(A,B)** The cardiac levels of triglyceride **(A)** and free fatty acid **(B)**. **(C,D)** The representative images of cardiac mitochondria **(C)** and quantitative analysis of lipid droplets **(D)**. **(E,F)** Western blot analysis of cardiac α-porin and PGC-1α **(E)**, and quantitative analysis of relative density **(F)**. **(G)** Real-time quantitative analysis of cardiac mitochondrial genes, including *Pgc-1*α, *Nrf1*, *Nrf2*, and *Tfam*. **(H–K)** Measurement of relative ATP production **(H)**, oxygen consumption **(I)**, citrate synthase activity **(J)**, and ratio of complex I/CS **(K)** in extracted cardiac mitochondria. Results are shown as mean ± SEM, and *n* = 6–8 mice/group. *p < 0.05, **p < 0.01, ***p < 0.001.

Mitochondrial function is especially important for the heart with high demands in energy, which is achieved through oxidative phosphorylation (Siasos et al., 2018). In contrast to A-ZIP/F-1 mouse hearts that had higher levels of triglyceride and free fatty acid in old age, administration of vaspin obviously decreased this lipid accumulation (Figures 3A,B, *p* < 0.001). The electronic microscopic analysis further found that the lipid droplet was hugely abolished in vaspin-treated A-ZIP/F-1 mouse hearts (Figures 3C,D, *p* < 0.001). The mitochondrial function was determined by mitochondrial biogenesis and activities (Siasos et al., 2018). Therefore, we first measured the mitochondrial quantity in A-ZIP/F-1 mouse hearts. As shown in Figures 3E,F, vaspin increased the expression of α-porin, a key mitochondrial inner membrane protein (*p* < 0.05). Real-time PCR analysis also found that vaspin increased the expression of mitochondrial genes, including *Pgc-1*α, *Nrf1*, *Nrf2*, and *Tfam* (Figure 3G, *p* < 0.01). Next, we analyzed the mitochondrial activities. As showed in Figure 3H, administration of vaspin increased cardiac mitochondrial ATP production to twofold (*p* < 0.01). The mitochondrial endogenous respiration activity, represented by oxygen consumption, was significantly increased in vaspin-treated cardiac mitochondria (Figure 3I, *p* < 0.001), whereas citrate synthase activity was also upregulated (Figure 3J, *p* < 0.001). Mitochondrial complex I is the major component of mitochondrial complexes and controls the oxidative phosphorylation (Nie et al., 2016, 2018b). Figure 3K shows that vaspin could increase the activity of complex I, compared with Veh-treated A-ZIP/F-1 mice.

### Implantation of Adipose Tissue Attenuates Lipoatrophy-Induced Cardiomyopathy Dependent on Vaspin Signaling

**FIGURE 4:**
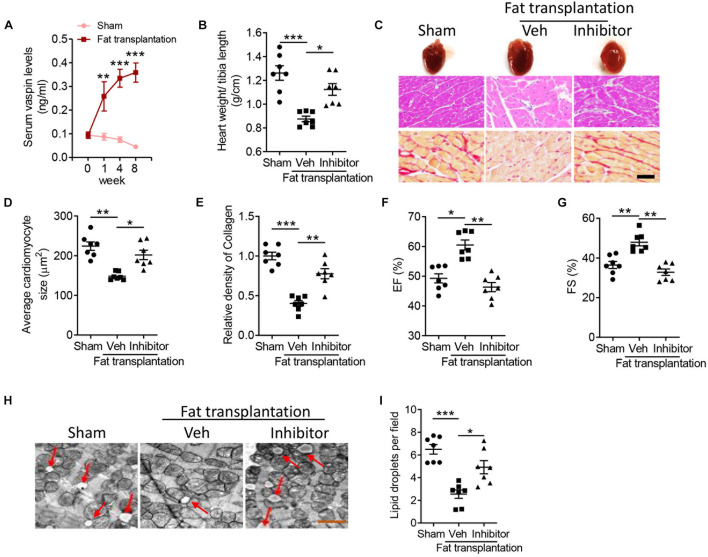
Blockage of vaspin reverses the cardioprotective benefits of fat transplantation in A-ZIP/F-1 lipoatrophic mice. Sixteen-week A-ZIP/F-1 mice were subcutaneously transplanted with 0.9 g of visceral fat from C57BL/6J mice and locally injected with 1 10^9^ lentivirus-encoding *vaspin* siRNA or control siRNA for 8 weeks. **(A)** The serum levels of vaspin in mice treated with recombinant vaspin protein for 0, 1, 4, and 8 weeks. **(B)** The ratio of heart weight/tibia length. **(C–E)** Representative images of whole hearts, and cardiac slides were stained with hematoxylin and eosin or Sirius red staining solution **(C)**, and quantitative analysis of average cardiomyocyte size **(D)** and relative density of collagen **(E)**. Scale bar = 20 μm. **(F,G)** Echocardiographic analysis of mouse cardiac EF **(F)** and FS **(G)**. **(H,I)** The representative images of cardiac mitochondria **(H)** and quantitative analysis of lipid droplets **(I)**. Results are shown as mean ± SEM, and *n* = 7 mice/group. *p < 0.05, **p < 0.01, ***p < 0.001.

Vaspin, named visceral adipose tissue-derived serpin, was first reported as an adipokine released from rat visceral adipose tissue (Liu et al., 2018). Besides, implantation of adipose tissues could improve lipoatrophy-induced metabolic phenotypes, such as hyperphagia and hepatic steatosis (Gavrilova et al., 2000). Therefore, we aimed to determine whether vaspin from adipose tissue could mediate cardiac benefits in A-ZIP/F-1 mice. After an 8-week intervention, there was no significant difference in adipose tissue structure (Supplementary Figure 3A) and gene expression of *adiponectin* or *A-fabp* (Supplementary Figures 3B,C), which indicated that the transplanted adipose tissue was alive. Interestingly, A-ZIP/F-1 mice transplanted with adipose tissue had higher circulating concentration of vaspin (Figure 4A). Furthermore, the gene level of vaspin in the liver or skin was not affected in mice transplanted with fat, which indicated that transplanted fat was the main source of circulating vaspin. Interestingly, transplantation of adipose tissue attenuated lipoatrophy-induced cardiac hypertrophy (Figures 4B–D) and fibrosis (Figure 4E). To address the critical role of adipose tissue-derived vaspin in cardiac remodeling, meanwhile, we locally injected lentivirus-encoding *vaspin* siRNA into the transplanted fat pad. Gene and protein analysis determined the effective silent ability of these lentiviruses in suppressing vaspin expression (Supplementary Figures 3E,F). Mice co-administrated with lentivirus-encoding *vaspin* siRNA exhibited an increased ratio of heart weight/tibia length (Figure 4B, *p* < 0.05), average cardiomyocyte size (Figures 4C,D, *p* < 0.05), and collagen contents (Figure 4E, *p* < 0.001). Echocardiographic analysis showed that transplantation of adipose tissue increased cardiac EF (Figure 4F, *p* < 0.05) and FS (Figure 4G, *p* < 0.01), but vaspin silence abolished these benefits (Figures 4F,G, *p* < 0.01). The left ventricle wall thickness, including LVAW, LVID, and LVPW, was decreased in mice transplanted with fat grafts, but increased after vaspin deficiency (Supplementary Table 1). The fat transplantation-decreased left ventricle mass was also abolished in A-ZIP/F-1 mice after administrated with lentivirus-encoding vaspin siRNA (*p* < 0.05). Furthermore, electronic microscopic analysis showed that implantation of fat grafts decreased the cardiac lipid droplets, but suppression of vaspin reversed the structural disorders (Figures 4H,I). Similarly, the cardiac lipids, including triglyceride and free fatty acid, were remarkably decreased in mice transplanted with adipose tissue (*p* < 0.001), but significantly reversed after silence of vaspin (Supplementary Figures 4A,B, *p* < 0.05). Silence of adipose tissue vaspin also increased cardiac levels of TNF-α, IL-1β, and NO, compared with vehicle-treated adipose tissue (Supplementary Figures 4C–E, *p* < 0.05).

### Contribution of Vaspin/AKT Signaling in Lipoatrophy-Induced Cardiac Remodeling

**FIGURE 5:**
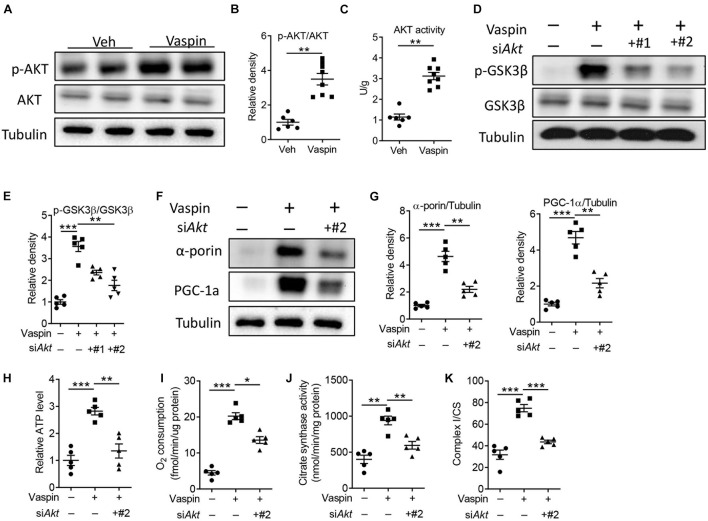
Vaspin improves cardiomyocyte mitochondrial function by upregulating phosphorylated AKT. **(A,B)** Sixteen-week-old A-ZIP/F-1 mice were treated with recombinant vaspin protein (100 μg/mouse) by osmatic pump for 8 weeks. Western blot analysis of cardiac phosphorylated AKT and AKT **(A)**, and quantitative analysis of relative density **(B)**. **(C)** The measurement of cardiac AKT activity by commercial kit. **(D–K)** Primary cardiomyocytes (1 10^6^) were incubated with 1 10^5^ viral particles encoding si*Akt* for 48 h (#1 and #2), then treated with recombinant vaspin protein (2.5 μg/ml) for 24 h. Western blot analysis of phosphorylated GSK3β and GSK3β **(D)**, and quantitative analysis of relative density **(E)**. Western blot analysis of α-porin and PGC-1α **(F)**, and quantitative analysis of relative density **(G)**. **(H–K)** Measurement of relative ATP production **(H)**, oxygen consumption **(I)**, citrate synthase activity **(J)**, and ratio of complex I/CS **(K)** in extracted cardiomyocyte mitochondria. Results are shown as mean ± SEM, and *n* = 6–8 mice/group or five independent experiments. *p < 0.05, **p < 0.01, ***p < 0.001.

A previous study had demonstrated that enhanced protein kinase B (AKT) signaling could protect against cardiac lipotoxicity (Zhao et al., 2020). In pancreatic β cell, administration of vaspin increased phosphorylation of AKT and improved cell function (Liu et al., 2017). Consistently, the phosphorylated AKT level was decreased in A-ZIP/F-1 mice, compared with C57 mice (Supplementary Figures 5A,B). Furthermore, the AKT activity was also decreased in lipoatrophic mice (Supplementary Figure 5C). Then, we measured the effect of vaspin on cardiac AKT signaling in A-ZIP/F-1 mice. As shown in Figure 5, administration of recombinant vaspin protein obviously upregulated the protein expression of cardiac phosphorylated AKT (p-AKT, Figures 5A,B, *p* < 0.01) and AKT activity (Figure 5C). Treatment of recombinant vaspin protein also dose-dependently increased the expression of p-AKT and p-GSK3β (Supplementary Figures 6A,B). To determine the critical role of AKT in vaspin-induced cardioprotective benefits, we silenced *Akt* in primary cardiomyocytes (Supplementary Figure 7A). As shown in Figures 5D,E, knockdown of *Akt* effectively suppressed the phosphorylation of downstream GSK3β (*p* < 0.01). In primary cardiomyocytes, silence of *Akt* suppressed vaspin-induced upregulation of α-porin or PGC-1α (Figures 5F,G, *p* < 0.01), but did not affect the expression of key mitochondrial genes in PBS-treated cardiomyocytes (Supplementary Figure 7B). Furthermore, administration of vaspin increased cardiac mitochondrial activities, including ATP production (Figure 5H, *p* < 0.001), endogenous respiration activity (Figure 5I, *p* < 0.001), citrate synthase activity (Figure 5J, *p* < 0.01), and complex I activity (Figure 5K, *p* < 0.001) in primary cardiomyocytes. However, consistent to mitochondrial biogenesis, blockage of AKT abolished these upregulation of mitochondrial activities (Figures 5H–K).

## Discussion

The present study, for the first time, addressed the biological role of vaspin in lipoatrophy-induced cardiomyopathy. In A-ZIP/F-1 lipoatrophic mice, reduction of cardiac function was closely correlated with circulating and adipose tissue vaspin levels. Replenishment of recombinant vaspin protein or fat transplantation effectively alleviated cardiac pathological remodeling in A-ZIP/F-1 mice. The upregulation of AKT activity was potential molecular mechanisms of vaspin in protecting against lipoatrophy-induced cardiac injuries.

Lipoatrophic patients, with a severe decrease in the amount of adipose tissue, are known to suffer from dyslipidemia, insulin resistance, and cardiovascular diseases (Reitman et al., 2000). Lipoatrophy, one of key profiles in patients with HIV, leads to consequent metabolic complications. HIV-related cardiovascular disease accounts for more than 2.6 million per year around the world. Previous studies have demonstrated that the risk of heart disease for HIV-infected patients was 1.5–2 times greater than healthy subjects (Klein et al., 2003). Researchers initially considered that the risk for cardiovascular disease was partially linked to antiretroviral therapy, a first-line strategy for curing HIV. However, recent studies addressed that the elevated incidence of heart disease might be related to lipoatrophy-induced chronic inflammation in cardiovascular system (Sekhar et al., 2004). Further studies showed that severe fat loss in anatomic sites induced dyslipidemia and lipotoxicity in peripheral tissues, including the heart, vascular, and liver. In A-ZIP/F1 lipoatrophic mice, there was a drastically reduced amount of fat (Moitra et al., 1998). These mice were prone to diabetes, with elevated serum insulin, lipids, and cardiac hypertrophy. It is necessary to uncover the molecular changes of lipoatrophy-induced cardiac pathological remodeling and explore the intraorgan crosstalk between adipose tissues and hearts.

In physiological status, the adipose tissues are not only the storage of neutral lipids but also endocrine tissues for producing various biological factors. Emerging studies have reported that bioactive adipokines, including RNA and protein secreted from adipose tissues, are involved in the development of cardiovascular disease. MicroRNAs and lincRNAs are potential therapeutic targets for cardiovascular diseases (Lucas et al., 2018; Pan et al., 2019). Adipose tissue-derived active protein participated in multiple metabolic networks. Imbalanced secretion of toxic cytokines contributes to the development of cardiovascular diseases, whereas beneficial factors have therapeutic benefits. Leptin, mainly synthesized and secreted from adipose tissues, was considered as one of the effective adipokines for lipoatrophy therapy (Oral et al., 2002). Mechanistically, leptin replacement therapy effectively suppressed inflammatory response and increased mitochondrial activities. Leptin also reversed lipodystrophy-induced insulin resistance and diabetic complications by regulating insulin signaling in mice (Shimomura et al., 1999). In A-ZIP/F-1 mice, leptin guaranteed the benefits of fat transplantation in improving metabolic homeostasis (Colombo et al., 2002). These clinical trials and animal experiments supported that adipokines bridged the interaction between adipose tissues and cardiovascular homeostasis in lipoatrophic status.

Vaspin, mainly secreted from adipose tissues, was exhibited as a predictor of heart diseases in several clinical trials. Zhou et al. (2019) reported that plasma vaspin concentration was decreased in patients with acute myocardial infarction. Circulating level of vaspin was negatively correlated with C-reactive protein or NT-proBNP, two key parameters of cardiac injuries. Administration of vaspin protected against cardiac pathological remodeling in response to multiple injuries. In diabetic rats, vaspin prevented myocardial damages (Li et al., 2019). Vaspin also alleviated myocardial ischemia/reperfusion injuries (Yuan et al., 2018). However, it was unknown whether vaspin was involved in the process of lipoatrophy-mediated pathological changes. In the present study, A-ZIP/F-1 lipoatrophic mice exhibited significant reduction in vaspin level during aging. Consistently, circulating level of vaspin was negatively associated with cardiac dysfunction, including reduction of cardiac EF and FS. More importantly, replenishment of vaspin improved cardiac structural remodeling and mitochondrial function. Combined with multiple previous reports, our findings provided evidence that targeting vaspin might be an effective strategy for alleviating lipoatrophy-induced cardiac injuries.

Vaspin, a serine protease inhibitor, made several direct biological effects on metabolic homeostasis. In white adipose tissue, vaspin modulated insulin action by specifically inhibiting its target protease KLK7 (Heiker, 2014). In pancreatic islet cells, administration of vaspin enhanced the phosphorylation of AKT/GSK3β signaling (Zhao et al., 2020). Vaspin also alleviated high glucose-induced cellular dysfunction induced by the PI3K/Akt pathway in endothelial progenitor cells (Sun et al., 2015). However, the molecular mechanisms of cardioprotective benefits of vaspin are still unknown. In the present study, we identified that cardiomyocyte AKT/GSK3β signaling might mediate the therapeutic effects of vaspin *in vivo* and *in vitro*. Also, AKT is a serine/threonine-specific protein kinase, which supports the possible involvement of protease inhibition of vaspin. Although it was unknown if there was direct binding consequence, our findings with previous reports could determine that vaspin/AKT signaling had therapeutic benefits in improving lipoatrophy-induced cardiac injuries.

Lipoatrophic A-ZIP/F-1 mice showed improvement in metabolic profiles after transplantation with adipose tissues (Colombo et al., 2002). Consistently, our transplanted fat also alleviated lipoatrophy-induced cardiac structural disorders and mitochondrial dysfunction, whereas blockage of fat vaspin abolished these cardioprotective benefits. Our novel finding indicated that supplementation of vaspin was a potential approach to combating lipoatrophy-induced cardiomyopathy.

## Conclusion

In conclusion, lipoatrophy emphasized that cardiac pathological remodeling is dependent on adipose tissue-derived vaspin. Replenishment of vaspin attenuated lipoatrophy-induced cardiomyopathy by modulating cardiac AKT/GSK3β activity. Therefore, targeting vaspin/AKT signaling was a potential strategy for combating lipoatrophy-induced cardiac injuries.

## Data Availability Statement

The raw data supporting the conclusions of this article will be made available by the authors, without undue reservation.

## Ethics Statement

The animal study was reviewed and approved by the Institutional Animal Care and Use Committee Guidelines of Harbin Medical University.

## Author Contributions

DZ, HZ, EZ, FW, YL, and WX performed the experiments and analyzed the data. JL and SL analyzed the data. WC, YW, and YP designed, discussed the study, and wrote the manuscript. All authors contributed to the article and approved the submitted version.

## Conflict of Interest

The authors declare that the research was conducted in the absence of any commercial or financial relationships that could be construed as a potential conflict of interest.

## Publisher’s Note

All claims expressed in this article are solely those of the authors and do not necessarily represent those of their affiliated organizations, or those of the publisher, the editors and the reviewers. Any product that may be evaluated in this article, or claim that may be made by its manufacturer, is not guaranteed or endorsed by the publisher.

## Supplementary Material

Supplementary Figure 1 Abnormal lipid profiles in serum and heart tissues of A-ZIP/F-1 lipoatrophy mice. **(A)** Fat mass percentage of 4-, 12-, and 24-week old A-ZIP/F-1 mice. The C57BL/6J (C57) mice were control group. **(B,C)** The cardiac levels of triglyceride **(B)** and free fatty acid **(C)**. Results are showed as mean ± SEM, and *n* = 6–8 mice/group. ^∗^*p* < 0.05, ^∗∗∗^*p* < 0.001.

Supplementary Figure 2 Administration of recombinant vaspin protein decreases cardiac inflammation and oxidative stress in A-ZIP/F-1 lipoatrophic mice. 16-week old A-ZIP/F-1 mice were treated with recombinant vaspin protein (100 μg/mouse) by osmatic pump for 8-week. **(A)** The circulating levels of vaspin after recombinant vaspin protein treatment. **(B)** The cardiac levels of vaspin protein. **(C–E)** Measurement of cardiac TNF-α **(C)**, IL-1β **(D)** and nitric oxide **(E)**. Results are showed as mean ± SEM, and *n* = 8 mice/group. ^∗∗^*p* < 0.01, ^∗∗∗^*p* < 0.001.

Supplementary Figure 3 The changes of transplanted fat and vaspin expression in different tissues in mice with transplanted fat. 16-week A-ZIP/F-1 mice were subcutaneously transplanted with 0.9 g visceral fat from C57BL/6J mice for 8 weeks. **(A)** H&E staining of transplanted visceral fat and control adipose tissues. **(B,C)** RT-qPCR analysis of adipose *adiponectin* **(B)** and *A-fabp* **(C)** levels (*n* = 6 mice/group). **(D)** RT-qPCR analysis of vaspin expression in liver and skin after fat transplantation for 8-week (*n* = 5 mice/group). **(E,F)** The efficiency of injected lentivirus on adipose vaspin expression. Analysis of gene **(E)** and protein **(F)** levels of vaspin in transplanted fat (*n* = 5 mice/group). Results are showed as mean ± SEM.

Supplementary Figure 4 Blockage of vaspin increased cardiac inflammation, oxidative stress and lipid deposits in A-ZIP/F-1 lipoatrophic mice transplanted with fat. 16-week A-ZIP/F-1 mice were subcutaneously transplanted with 0.9 g visceral fat from C57BL/6J mice, and tail-veil injected with 1 10^9^ lentivirus encoding *vaspin* siRNA or control siRNA for 8 weeks. The cardiac levels of triglyceride **(A)**, free fatty acid **(B)**, TNF-α **(C)**, IL-1β **(D)**, and NO **(E)**. Results are showed as mean ± SEM, and *n* = 7 mice/group. ^∗^*p* < 0.05, ^∗∗^*p* < 0.01, ^∗∗∗^*p* < 0.001.

Supplementary Figure 5 The changes of cardiac AKT activity in A-ZIP/F-1 lipoatrophy mice. **(A)** Western blot analysis of cardiac levels of phosphorylated AKT and total AKT in 4-, 12-, and 24-week old A-ZIP/F-1 mice and C57BL/6J (C57) mice. **(B)** Quantitative analysis of relative density. **(C)** The measurement of cardiac AKT activity by commercial kit. A-ZIP/F-1 mice aged 24-week were sacrificed, and the cardiac AKT activity was analyzed. Results are showed as mean ± SEM, and *n* = 6–8 mice/group. ^∗∗^*p* < 0.01, ^∗∗∗^*p* < 0.001.

Supplementary Figure 6 The effects of vaspin on AKT/GSK3β activity. 1 10^6^ primary cardiomyocytes were treated with different concentrations of recombinant vaspin protein (0, 0.05, 0.25, and 2.5 μg/ml) for 24-h. Western blot analysis of p-AKT, AKT, p-GSK3β, and GSK3β **(A)**, and quantitative analysis of relative density **(B)**. Results are showed as mean ± SEM, and *n* = 5 independent experiments. ^∗∗^*p* < 0.01.

Supplementary Figure 7 The efficiency of lentivirus encoding siAkt in cardiomyocytes and downstream mitochondrial changes. **(A)** 1 10^6^ primary cardiomyocytes were incubated with 1 10^5^ viral particles encoding si*Akt* for 48-h (#1 and #2), and western blot analysis of AKT expression. **(B)** The mitochondrial gene expression in cardiomyocytes treated with lentivirus encoding siAkt (#2). Results are showed as mean ± SEM, and *n* = 5 independent experiments.

Supplementary Table 1 Echocardiographic analysis of mice transplanted with adipose tissue with or without lentivirus encoding *vaspin* siRNA.
