# Supplementary material for: Vaspin Mediates the Intraorgan Crosstalk Between Heart and Adipose Tissue in Lipoatrophic Mice
Source: Front Cell Dev Biol. 2021 Sep 24;9:647131. doi: 10.3389/fcell.2021.647131 (PMC8497826; doi:10.3389/fcell.2021.647131)
Supplement: Supplementary Figure 1 — Abnormal lipid profiles in serum and heart tissues of A-ZIP/F-1 lipoatrophy mice. (A) Fat mass percentage of 4-, 12-, and 24-week old A-ZIP/F-1 mice. The C57BL/6J (C57) mice were control group. (B,C) The cardiac levels of triglyceride (B) and free fatty acid (C). Results are showed as mean ± SEM, and n = 6–8 mice/group. ∗p < 0.05, ∗∗∗p < 0.001. [file Presentation_1.PPTX]

## Slide 1
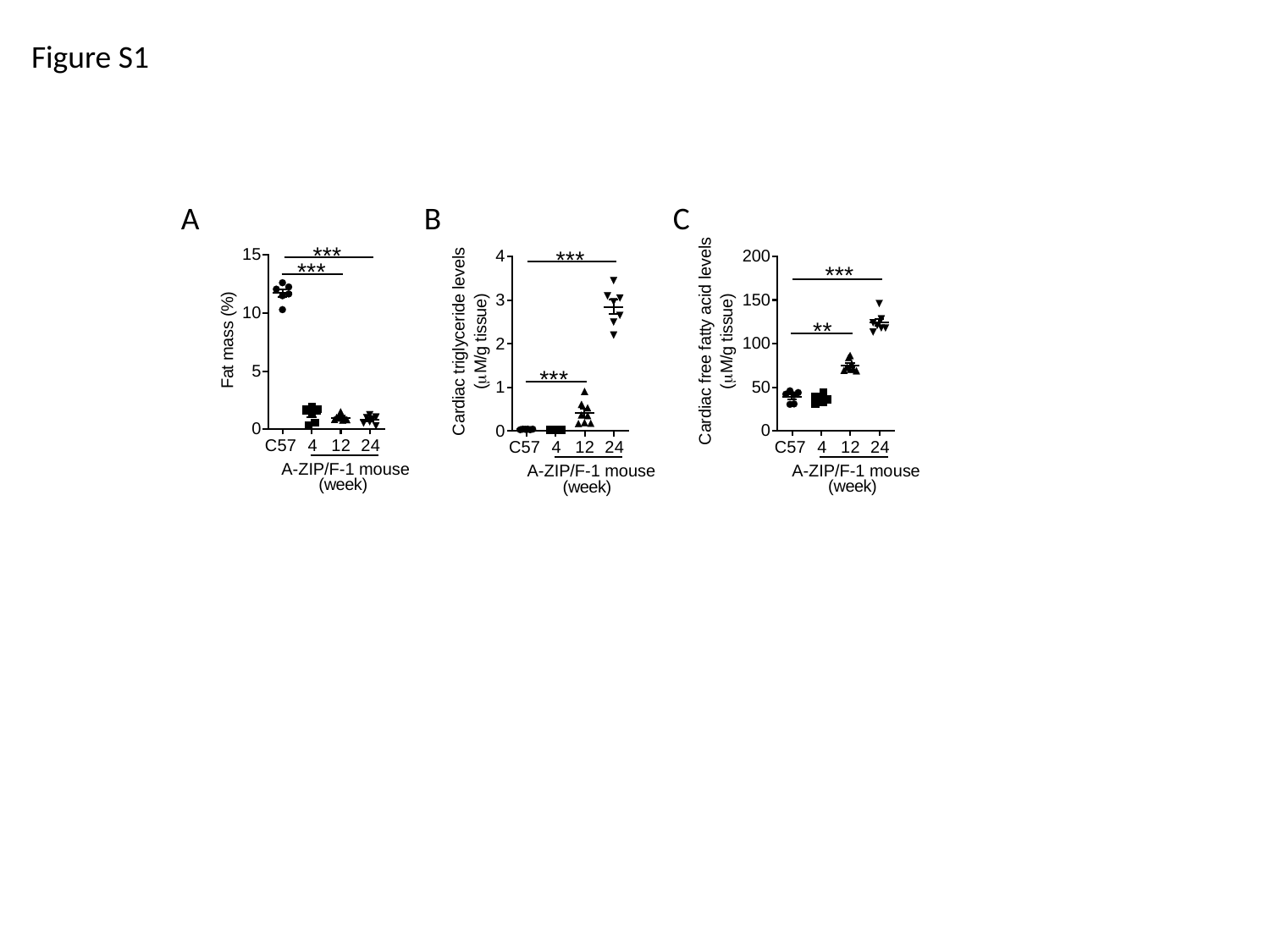

Figure S1
A
C
B

## Slide 2
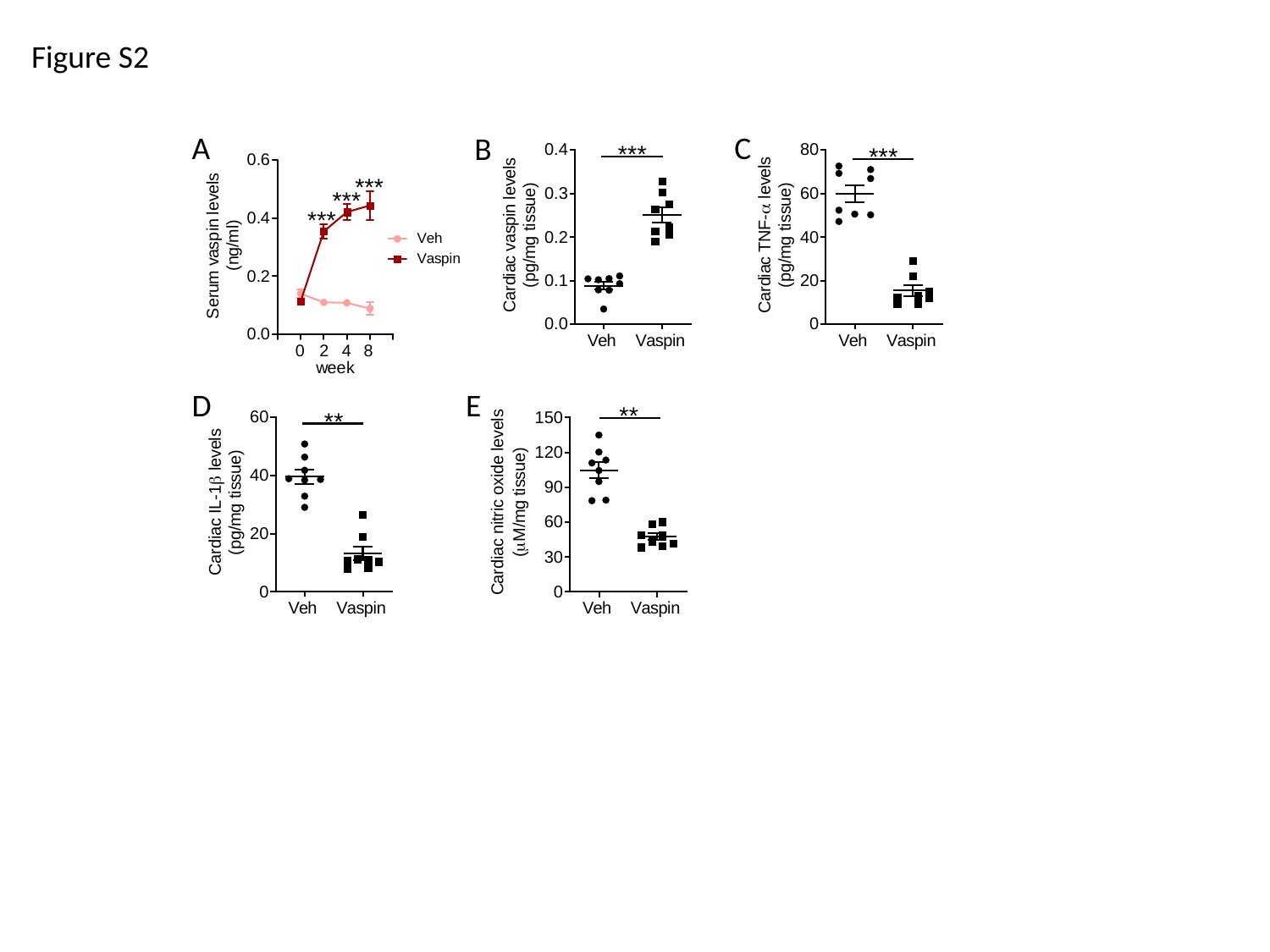

Figure S2
A
C
B
D
E

## Slide 3
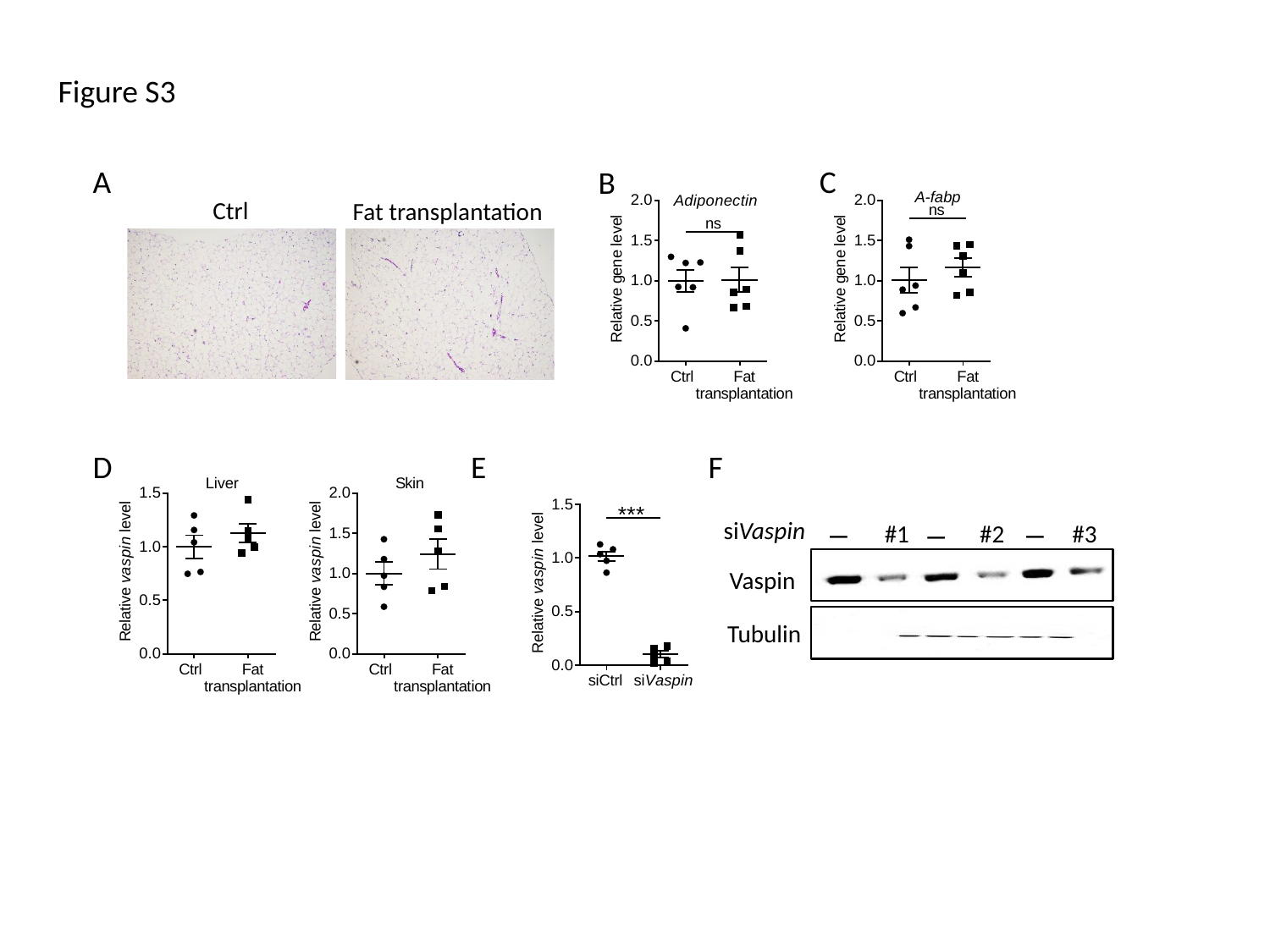

Figure S3
A
C
B
Ctrl
Fat transplantation
D
E
F
_
_
_
siVaspin
#1
#2
#3
Vaspin
Tubulin

## Slide 4
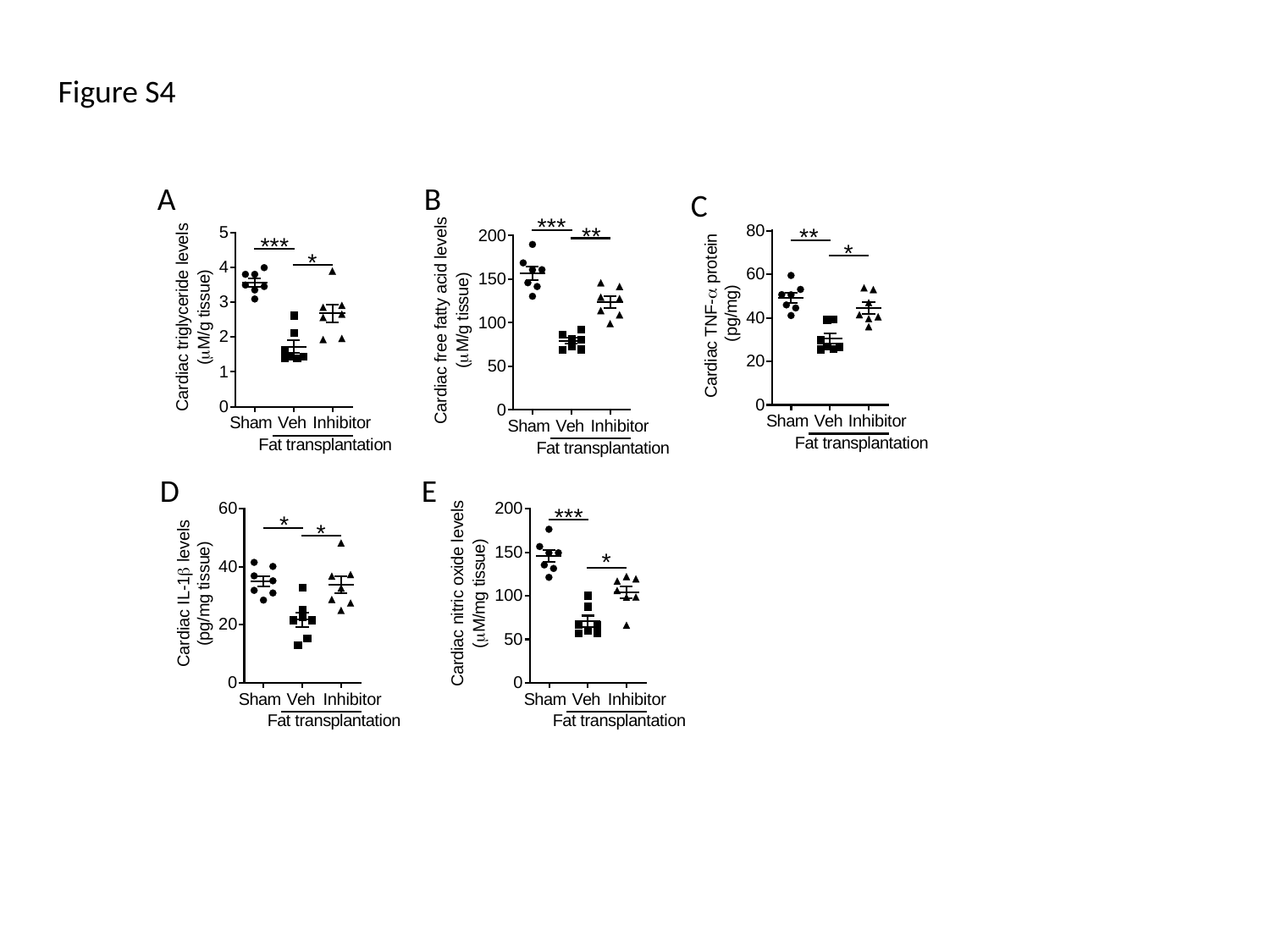

Figure S4
A
B
C
D
E

## Slide 5
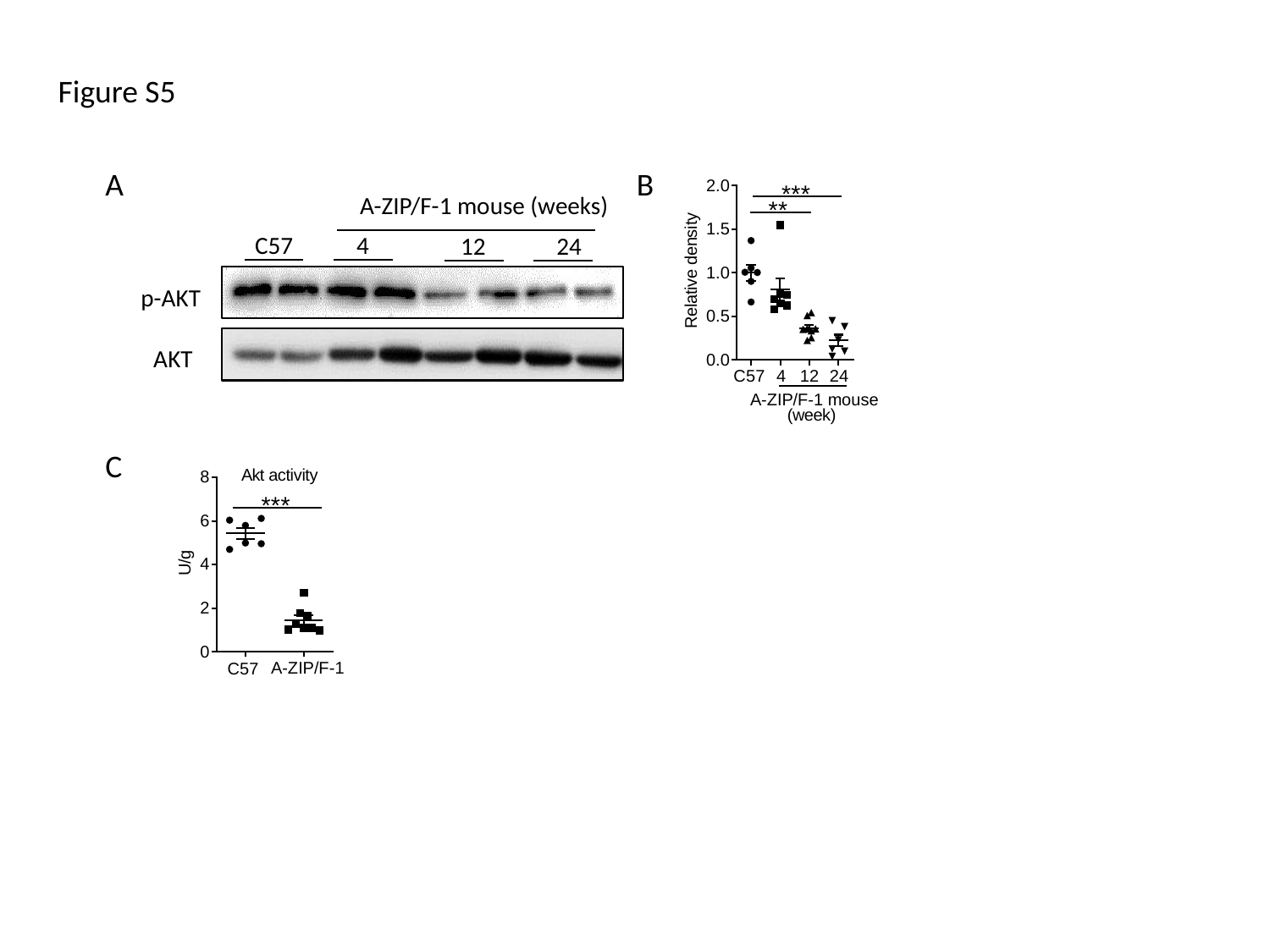

Figure S5
B
A
A-ZIP/F-1 mouse (weeks)
4
C57
24
12
p-AKT
AKT
C

## Slide 6
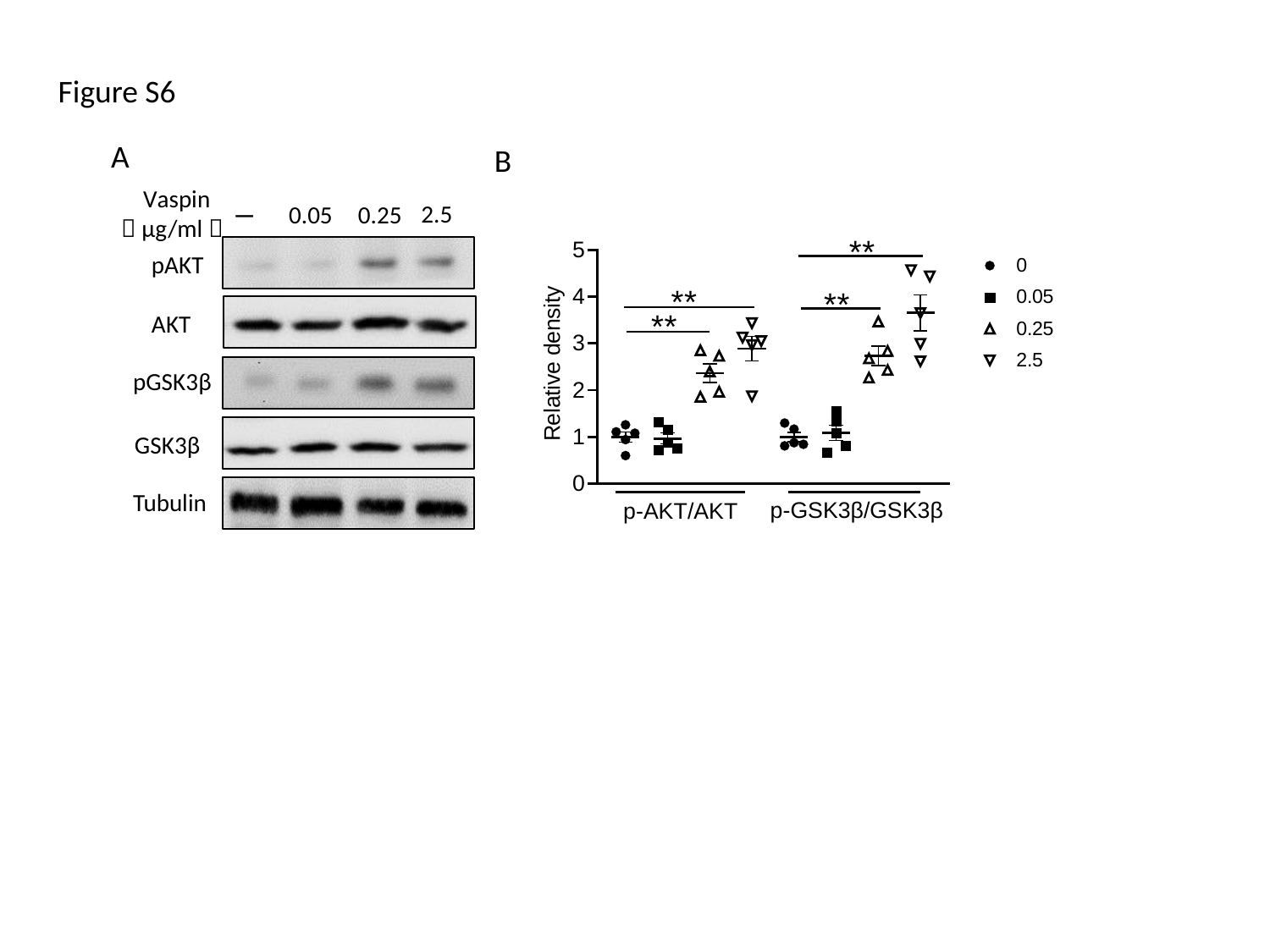

Figure S6
A
B
_
 Vaspin
（µg/ml）
2.5
0.05
0.25
pAKT
AKT
pGSK3β
GSK3β
Tubulin

## Slide 7
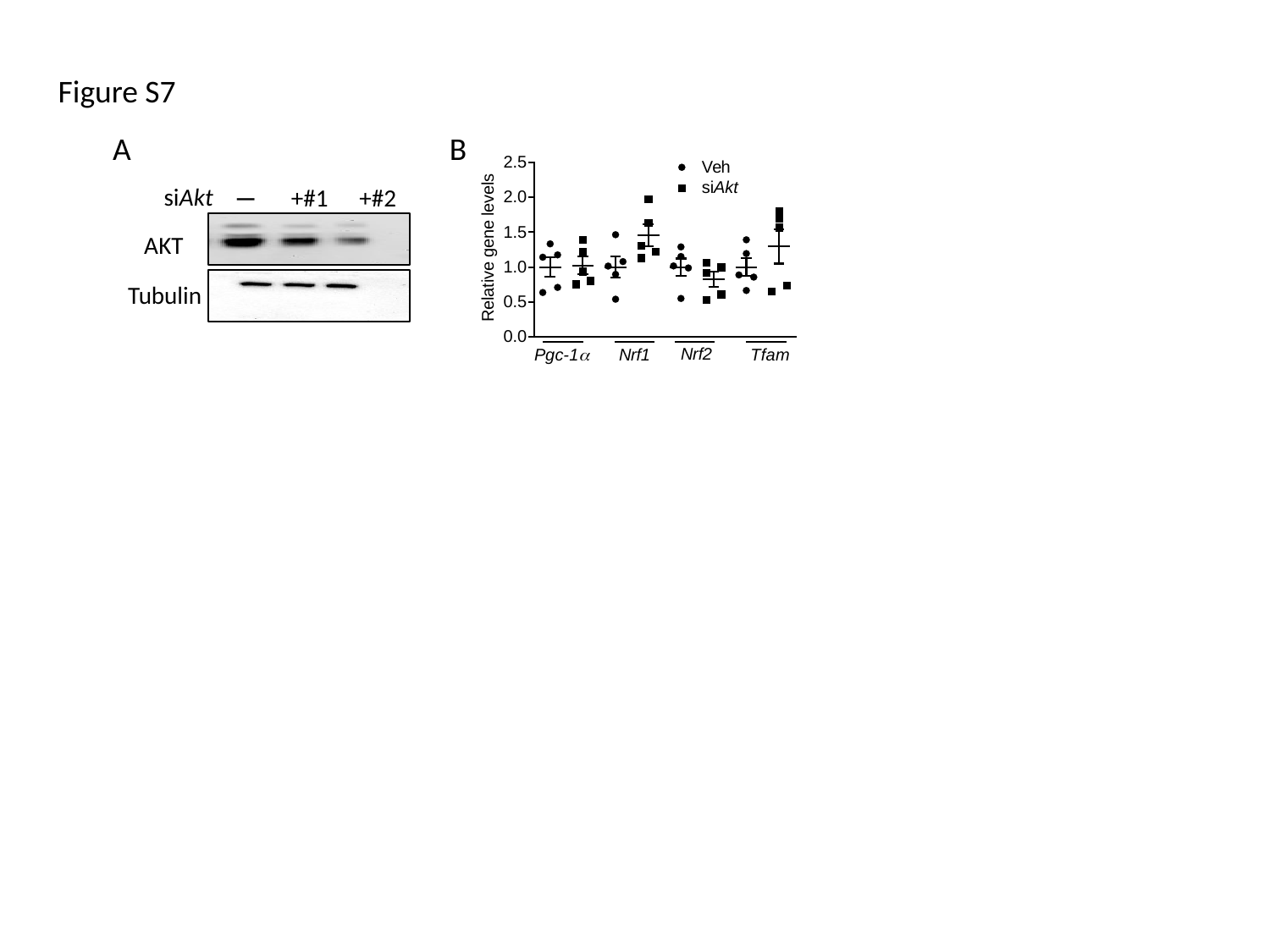

Figure S7
A
B
_
siAkt
+#1
+#2
AKT
Tubulin
