# Supplementary material for: Vaspin Mediates the Intraorgan Crosstalk Between Heart and Adipose Tissue in Lipoatrophic Mice
Source: Front Cell Dev Biol. 2021 Sep 24;9:647131. doi: 10.3389/fcell.2021.647131 (PMC8497826; doi:10.3389/fcell.2021.647131)
Supplement: Supplementary Table 1 — Echocardiographic analysis of mice transplanted with adipose tissue with or without lentivirus encoding vaspin siRNA. [file Table_1.DOCX]

Table S1. Echocardiography analysis of mice transplanted with fat with or without lentivirus encoding *vaspin* siRNA

| Variables | Sham | FT + Veh | FT + Inhibitor |
| --- | --- | --- | --- |
| Heart rate (beats/min) | 483 ± 39 | 461 ± 16 | 472 ± 30 |
| LVAW;d (mm) | 1.31 ± 0.28** | 0.89 ± 0.34 | 1.27 ± 0.22** |
| LVID;d (mm) | 4.44 ± 0.52*** | 3.63 ± 0.29 | 4.10 ± 0.38** |
| LVPW;d (mm) | 1.23 ± 0.21** | 0.91 ± 0.10 | 1.19 ± 0.23* |
| LVAW;s (mm) | 1.74 ± 0.27** | 1.31 ± 0.32 | 1.68 ± 0.24* |
| LVID;s (mm) | 2.82 ± 0.35*** | 1.98 ± 0.42 | 2.37 ± 0.29* |
| LVPW;s (mm) | 1.62 ± 0.21** | 1.19 ± 0.37 | 1.49 ± 0.12* |
| LV mass (mg) | 158.89 ± 27.23** | 118.77 ± 21.88 | 143.14 ± 16.34* |

Data are means ± SEM. FT + Veh, fat transplantation + lentivirus encoding control siRNA; FT + Inhibitor, fat transplantation + lentivirus encoding *vaspin* siRNA; d, diastolic; s, systolic; LVAW, left ventricular end anterior wall thickness; LVID, left ventricular internal dimension; LVPW, left ventricle posterior wall thickness; LV mass, left ventricle mass. * p < 0.05, ** p < 0.01 and *** p < 0.001.
